# AI ethics, accountability, and sustainability: revisiting the Hippocratic oath

**DOI:** 10.1080/17453674.2019.1682850

**Published:** 2019-10-30

**Authors:** Li Felländer-Tsai

**Affiliations:** Division of Orthopaedics and Biotechnology, CLINTEC, Karolinska Institutet, Sweden

Artificial intelligence (AI) embedded in healthcare technologies creates opportunities and great expectations. However, new interactions between man and machine arise and must be addressed (Lancet 2017). Unfortunately, medical education and training at all levels lag behind the advancement of technology. New knowledge in healthcare and medicine emerges so rapidly that when newly graduated doctors have completed their specialist training, much knowledge has already become obsolete. In order to ensure quality and patient safety and to benefit from the positive opportunities that AI-based technology creates, new insights, investments, and focus are pivotal.

When new technologies enter the medical and healthcare stage, physician training must ensure that these are used in a safe, effective, and evidence-based manner. In orthopedics and traumatology, the potential for more efficient and precise diagnostics with AI-based image interpretation has been widely identified. However, in medicine in general AI is often mentioned as a gray area and thus requires special attention in order to ensure ethics, fairness, accountability, and sustainability.

The availability and trust in medical decision support tools based on AI is increasing, despite the fact that education and training in how these potentially black boxes function is lagging behind. The initially somewhat restrictive attitude in the healthcare sector is now undergoing rapid expansion. Large amounts of data are considered the new “gold” and are being embraced by stakeholders at all levels in healthcare and medicine. This pinpoints the need for education, as well as critical scrutiny and discussion of the ethical implications regarding data management and AI in healthcare and medicine. Recent media reports of data breaches on a number of social media platforms have shed light on these emerging challenges.

When designing algorithms for decision support, measurements, references, and validation must be carefully defined in collaboration with medical experts. Systematic monitoring and validation of the algorithms in close collaboration with experts is also necessary. Bias and confounders must be managed because the origin of data on which the algorithms are based can lead to erroneous interpretations and potential damage (Box).

From an ethical perspective, understanding, insight, and transparency regarding potential self-learning and automated decision support tools are necessary before they can be commercialized and scaled up (Larsson 2018). The level of explainability and transparency required for the responsible physician to trust the increasingly autonomous decision support tools must also be defined. Transparency is necessary for regulation of commercial AI products in order for them to be able to receive appropriate learning feedback, but also to meet society’s need for accountability when new products lead to potential unintentional, unwanted, or unexpected outcomes. Responsibility and accountability must be discussed and clarified. There must be no doubt about who should be held accountable when AI represents a third party besides the responsible physician and the patient in healthcare. Is AI as a partner and member of the healthcare team feasible in clinical decision-making and execution of tasks?

Examples of unintended consequences or misuse of AI

- Ethnic bias in apps using face recognition for detection of genetic ­disorders
- Machine learning applications in image databases that not onlyreproduce but reinforce gender bias
- Ethnic bias in algorithm-based practices for predicting recidivism in crime
- Unethical and questionable AI-based marketing practices based on user data
- Misinterpretations of surroundings or data leading to fatal accidents in AI-based navigation systems/robotics/autonomous vehicles

A couple of examples highlighting the challenges to applied AI in the healthcare sector are applications intended for patients and doctors as well as robotics and systems collecting big data while they are used. A clear ethical dilemma may arise if the user is unable to understand or interpret the user instructions and product description. Even though there is great potential for efficiency and scalability by creating these kinds of platforms in healthcare, there is a risk of bias and confounders, as well as unintentional use and interpretation of data. The risk of under-use, misuse, and overuse are potentially serious matters in terms of accountability and ethics.

## The balance between access to data and the right to privacy

The large amounts of data required to develop AI create value conflicts between regulation and laws that protect the integrity of both patient and physician information—an important part of medical ethics—and the access to large amounts of data that the development of AI tools requires. We must also be able to ask the question and discuss how much consideration should be given to patients and physicians when their information is released to train AI. Tension at the crossroads of innovation and individual integrity may evolve. Ethics has always been a central part of both medical science and healthcare practice. A new chapter in relation to new technology and AI is undoubtedly needed. Pure enthusiasm, bounty hunting, and the notion of value creation must not challenge the interests of patient and physician integrity. The frequently paraphrased line in the Hippocratic oath “I will abstain from all intentional wrong-doing and harm” must be revisited. Unintentional harm definitely must not be overlooked.

Li Felländer-Tsai *Division of Orthopaedics and Biotechnology, CLINTEC, Karolinska Institutet, Sweden* *Email:* Li.Tsai@ki.se
